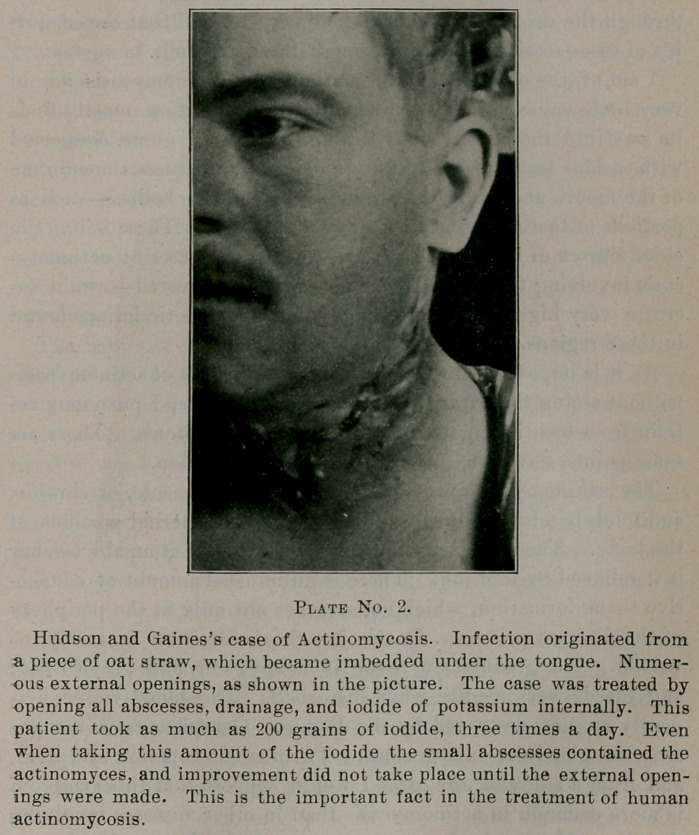# The Diagnosis and Treatment of Human Actinomycosis, with Report of Two Cases

**Published:** 1901-12

**Authors:** William H. Hudson

**Affiliations:** Rose Hill Sanatorium, Columbus, Ga.


ATLANTA

### Journal-Record of Medicine.

**Successor to Atlanta Medical and Surgical Journal, Established 1855, and Southern Medical Record, Established 1870.**

VOL. III. DECEMBER, 1901. NO. 9.

BERNARD WOLFF, M.D., EDITOR, Nos. 319–20 Prudential.

M. B. HUTCHINS, M.D., BUSINESS MANAGER, No. 64 Marietta St.

Published Monthly.

### *ORIGINAL COMMUNICATIONS.*

### THE DIAGNOSIS AND TREATMENT OF HUMAN ACTINOMYCOSIS, WITH REPORT OF TWO CASES.

BY WILLIAM H. HUDSON, M.D.,
ROSE HILL SANATORIUM, COLUMBUS, GA.

It is not the disease that is new, but our knowledge of it. How common it is for the people to speak of the present frequency of appendicitis, a disease of which they never heard a few years ago. Even physicians speak of it in the same way. When I began the practice of medicine, fourteen years ago, I had not heard of appendicitis. But no one will say that appendicitis did not exist then. Now every physician finds this disease.

It is said that Sir William Jenner taught that there had been no case diagnosed as floating kidney which proved to be such at the post mortem. And a case he exhibited as a floating kidney proved to be a uterine fibroid with a long pedicle. Cases of floating kidney are common enough to-day. The same may be said of acromegaly, myxedema, osteitis deformans, and other so-called rare diseases. All these diseases existed before, but our eyes had not been opened by the Pagets, Gulls, Maries, Ords and the others.

From the few cases of human actinomycosis reported in this country it certainly would appear that this is a rare disease. It is indeed very much rarer than some diseases; but when we come to look for it, and to know how to look for it, it will, I think, suffer the fate of other so-called rare diseases—it will not be so rare after all.

I am sure that scattered over this broad country there are many, perhaps thousands of cases of human actinomycosis; and many of them will be brought to light as the physicians come to look for them. A proof of this statement appears in the fact that most of the reported cases are from medical centers—places naturally, where the most accurate and best observers are at work. New York gives 17 cases; Chicago, 14; Baltimore, 5; Boston, 2; Brooklyn, 2; Cleveland, 2; Milwaukee, 2; Philadelphia, 3; Cambridge, Mass., 1; Buffalo, 1; Lucerne county, Pa., 1, reported from Philadelphia; Waterford, Va., 1, reported from Baltimore; Dubuque, Iowa, 1; Osceola, Wis., 1; St. Paul, Minn., 1; Minnesota, 1; Denver, 1; LaFayette, Ala., 2. Figures taken from Ruhrah. There have been other cases reported since the publication of his article, but I have had no opportunity to collect them.

It was thought, at one time, that the disease was very frequent in the neighborhood of Berlin, also in Austria; but this apparent frequency was only due to the observers looking out for it, and recognizing the cases when they were seen. On the contrary, in France, and some other countries, the disease was considered very infrequent. But as a knowledge of actinomycosis increased the cases came to light in various localities. One man, Poncet of Lyons, reporting 12 cases which he observed himself. So, with these facts in view, it seems unquestioned that human actinomycosis is of broad distribution; and is of comparative frequency.

*History.*—As I wish this to be a practical article, I will not go very lengthily into the history of our knowledge of human actinomycosis. It will suffice to say that the organism was first found in animals suffering with the disease which was known as osteosarcoma, by Rivota, in 1868. The great surgeon, von Langenbeck, had seen the organism in 1845. Bollinger, in 1877, made the first thorough study of actinomycosis; and had Harz, the botanist of Munich, to make a study of the organism. Harz gave the name actinomycosis rayfungus to the organism.

In 1878, J. Israel reported two cases of human actinomycosis. The next year Ponfick discovered the organism in a case of prevertebral abscess; and was the first to suggest the identity of the disease in animals and man. Since that time actinomycosis has been studied carefully by many observers in various countries.

*The Parasite.*—I will not attempt an amateur description of the parasite, and will therefore not enter deeply into the consideration of the biological properties of the different organisms which have been described. "It is evident, from recent investigations, that the micro-organism of actinomycosis must be included among the streptothrices; it is a pleomorphic and branching organism. Among its different forms are to be included cocci, rods (bacilli) and branching filaments. It has been proposed by Rossi-Doria to call the organism streptothrix actinomyces—a suggestion which it would be well to follow in the present somewhat confused state of its nomenclature. It should be borne in mind that it belongs properly, not to the fungi, but to the bacteria.

"The characteristic rosette-like configuration of the parasite depends in part upon degenerative changes within it. The swollen flasklike extremities are involutionary or degenerative products of retrogade metamorphosis. In successfully stained specimens these swellings may be seen to sit upon, like a hood, the preserved filaments. Hence in rapidly growing forms this appearance may be quite entirely wanting, the micro-organism now appearing as spheres and branching threads. The swellings, too, are not very durable structures; in pus they may disappear upon standing for a few days. In general it may be said that where much new tissue (productive inflammation) is being formed, there the organism is growing least rapidly and undergoing most degeneration, whereas where the organism increases with most rapidity, there the destruction of tissue (softening) is most pronounced and the degeneration of the parasite least. In man suppuration is more common as a feature in actinomycosis than in cattle, where the new tissue formed tends to produce large tumor-like masses. Whether, as Baumgarten suggest, the difference depends upon the coexistence in man of a pyococcal infection with the actinomyces, while in cattle the infection is a monoinfection, further study directed to the solution of this interesting problem is needed."—*Flexner*.

That we may be fully awake to the future possibilities in the pathology of actinomycosis, it is well, I think, to discuss, briefly, some of the recent views as to the parasites which may produce the clinical symptoms.

In reviewing the recent literature of actinomycosis and of the streptotricheæ, one is brought to believe that the term actinomycosis includes several species, or, perhaps, genera of micro-organisms. Hitherto there has been a tendency to accept the demonstration in the tissues or discharges of club-shaped organisms with a rosette arrangement as sufficient proof that the pathological condition was due to the presence of one definite organism, the actinomyces. Recent researches have demonstrated that such inference is unsafe.

It has been shown by several observers that widely different organisms in certain stages of their development exhibit filamentous forms with club-shaped swellings on their ends arranged in rosette fashion.

Further observations are necessary before accepting as final the results obtained by Freidrich and Babes and Levaditi, who inoculated cultures of the bacilli tuberculosis into the carotid artery and beneath the dura mater in rabbits, and found that the bacilli had not only branched, but had also developed into rosettes, resembling the "Drusen" of actinomyces; but certainly the observations are very interesting ones.

It seems inevitable that the words pseudo-actinomycosis, as well as pseudo-tuberculosis should come into our terminology, and by these terms we are to understand that we may find the clinical features of these diseases without finding the characteristic organisms.

We should keep these facts in mind, and when we fail to find the typical actinomyces we should be on the lookout for other organisms as the cause of the disease.

*Methods of Infection.*—It is very important that the people be taught that there is such a disease as actinomycosis. And it is equally important that they be taught the different ways through which human beings may become infected with the disease. It is thought that the organism loses its virulence in passing through animals. But there are cases reported which evidently became infected by driving a horse with "lumpy jaw"; and by treating cattle affected with the disease. There is only one case reported of infection from man to man.

Naturally, animals infected with actinomyces would be obnoxious as food, and of course should not be eaten; nor should the milk from cattle suffering with the disease be used as food. Although the organism has never been found in milk, Bollinger was of the opinion that his case of brain actinomycosis was due to the use of unboiled milk—as the patient subsisted mostly on this article of food during the last year of his life.

Next, we come to consider the most important and most interesting mode of infection: namely, from grain, its stalk and grasses. This mode of infection is fully established, and may occur in two ways. First: By breathing in the infected dust from the grain and its stalks during threshing. Second: By direct infection from the grain, or some other portion of the plant.

Numerous observations have been made in which portions of grain, or the sheaths of grain or straw, have been found in the actinomycotic lesion. In other cases, the patients were in the habit of using straw as tooth-picks, or of chewing raw grain. Poncet reports the case of an old woman who carried wisps of straw in her mouth with which to tie up vines. And Leith reports a case where the patient carried grain in his pockets to chew on at odd moments.

The second case reported here by myself, is conclusive, as the patient himself stated, when I first saw him, that the disease under his tongue was due to getting a piece of oat straw under his tongue, which he could not get out, and which was the starting point of his disease.

It is important to know that the parasite may grow upon most forms of grain and perhaps on various grasses; and that the people should be taught to cease chewing raw grain, and that they

PLATE No. 1.

Hudson's case of Actinomycosis. The disease involved the tissues of the back, and later the lungs. The sinuses were cleared out with sharp curette, and iodide of potassium was given internally; but the man died.

This case was reported in *Annals of Surgery*, November, 1897. The iodide of potassium was not used as I would now advise its use.

should stop the use of all portions of grain stalks, hay and grasses in their mouths.

Curiously, enough, a number of recent observers incline to the opinion that there may be some connection between the streptothrix actinomyces and the tubercle bacilli; and suggest that the tubercle bacilli may have their origin among the cereals—both from the same source.

*Diagnosis.*—It is worth our while to consider, briefly, the seat of the primary lesion as actinomycosis appears in cases reported from the United States; and for practical purposes this applies to all reported cases. The lower jaw, mouth and throat, 21; upper jaw and cheek, 9; bronchi, lungs, 12; intestinal tract, 17; skin, 3; breast, 1; and arm, 1. Of course, actinomycosis may attack any portion of the human body where the parasite finds an entrance into it. But, as by far the greatest chance of infection is through the mouth and bronchi, it is very natural that the majority of cases receive infection through these channels.

I am of the opinion that all statistics of actinomycosis are of very little value, for, it is evident that the infection mostly finds its way into the human body through ulcerated gums, connected with carious teeth, and through injuries of the mucous membrane of the mouth and pharynx, produced by foreign bodies—such as portions of tooth-pick, grain, straw and grass. These being the chief sources of infection, I think—should all cases of actinomycosis involving the regions of the jaws be discovered—would increase very highly the percentage of actinomycotic lesions found in these regions.

As it is impossible to make a clinical diagnosis of actinomycosis without seeing the parasite under the microscope, I purposely refrain from mentioning many of the clinical symptoms. There are some points, however, to which I will call attention.

The actinomycotic process is substantially subacute, or chronic, and tends to advance through sinuses to the external portions of the body. The pain is not the characteristic pain of an abscess, nor is it relieved by incision. There is an unusual amount of connective tissue formation, which ends rather abruptly at the periphery of the diseased process. In the jaw the bone itself is rarely involved. The granulation tissue is flabby, soft, and grayish red; and bleeds very readily when disturbed. The blood-vessels evidently are not destroyed by the disease. Conspicuous is the absence of glandular enlargement. Trismus is a common symptom, and from a study of the cases I would suppose that this symptom is more common in actinomycosis than in other diseases involving the jaws. Metastasis, from the absence of glandular enlargement, seems to occur through the blood current.

Chronic, recurrent abscesses, without necrosis—more or less painless, peculiar, crater-like sinuses, with circumscribed openings; subcutaneous abscesses, with firm margins—not connected with tuberculous glands, peculiar wooden hardness of the tissues, and, yet with a certain elasticity, would certainly suggest the disease.

About the alveolar process any ulcer or abscess should be looked upon with suspicion and great care taken to make a positive diagnosis. There can be no doubt that, relatively, a very large percentage of these lesions are actinomycotic, and no doubt many of them get well from bursting or from simple incision.

Actinomycosis cases involving the thorax and abdomen would be impossible to diagnose from the clinical symptoms. Here we are dependent upon the microscopical examination of the sputum, pleural effusions and intestinal discharges.

It should be remembered that the bones may be involved in actinomycosis—the favorite being the spinal column, first, and secondly, the sternum and ribs. Of course it is not impossible for any other bones of the body to become affected.

The habits of the patient, or some incident connected with the onset of the disease, may throw some light on the diagnosis. For instance, in case No. 2, here reported, the diagnosis of actinomycosis was made and the treatment begun several weeks before the parasite was found; the diagnosis being mainly based upon the patient's statement that an oat straw under his tongue had caused the disease, and from the presence of trismus, sudden onset, difficulty in swallowing, and absence of glandular involvement; and the peculiar wooden hardness which existed under the left side of his lower jaw, and the characteristic pain.

Of course we could not be absolutely positive of the diagnosis until the parasite was found; and this is the point which I wish especially to stress, that the parasite must be diligently looked for in every case which may possibly be one of this disease.

In case No. 1, reported here, numerous examinations were made of the pus from the open sinuses before the parasite was found. When a small, fluctuating abscess was opened the sulphur-like granules were seen in abundance. In case No. 2 the first pus which the patient brought to me, which he obtained from under his tongue, showed numbers of the organisms in question.

To make a positive diagnosis a microscopical examination of the discharge is necessary; but the mere presence of the so-called sulphur-like granules, examined by the naked eye and under a low power of the microscope, are not conclusive.

Small, round masses of fibrin, or tubercular *dèbris*, from the mouth or adjacent regions may very closely resemble a colony of actinomyces. Round masses of mouth bacteria, also, may closely resemble a colony. The latter, under a low power of the microscope, may present a radiating appearance and require a more careful examination with a higher power to show that it consists of masses of bacilli and thick, non-branching filaments of leptothrix buccalis.

In examining for actinomycosis care should be used to stop all bleeding before the abscess is opened. The pus from an abscess, freshly opened, is very much clearer than ordinary pus. It may be slightly blood-tinged and is somewhat sticky. It is best to catch the pus in a test-tube, or on a microscopical slide, as it runs out of the abscess. The granules are then plainly seen, and vary in size from a mere speck to a large size pin-head. They are round, irregularly oblong, or irregularly kidney-shaped. Gray, or grayish-yellow in color—the center being usually a little darker. It is easy to pick the granules out of the discharge, when they may be examined in glycerine. The fluid should be examined at once, before the blood has clotted.

There are certain other diseases which may be confused with actinomycosis, but it is thought best not to mention them here.

*Treatment.*—To a clinician the diagnosis and the treatment of a disease go hand in hand, and are its most important aspects. He must necessarily leave a certain amount of theoretical investigation to the laboratory scientists of medicine. It is a curious and interesting fact that as our ability to cure a disease increases, the number of remedies offered rapidly diminishes. How much variation would there be in the answers given by the members of this body to the questions. What remedies would you use in the treatment of malaria, of syphilis, of myxedema? I am sure the answers would be practically the same. This certainly would not be the case should I ask the treatment of some disease with which we are as yet unable to battle.

In the treatment of human actinomycosis I think we have reached that point of efficacy in treatment where the remedies are reduced to two :

The surgical and one drug.

One or two other drugs might be mentioned which have been given patients who recovered.

The medical means is iodide of potassium.

*Surgical.*—It depends to a great extent upon when the diagnosis of actinomycosis is made, as to the exact treatment which it will be best to follow. If a small abscess, about the alveolar process is found to be actinomycotic—thorough surgical means would certainly be indicated—care being used to remove all the disease, and then to pack the cavity with iodide of potassium gauze. Iodoform gauze would, perhaps, be just as efficient. If the actinomycotic process is more extensive it would be well to make an *external opening* from the disease *to the skin*; the entire channel to be gently packed with the gauze. When we consider the action of the iodide of potash, which I shall speak of more fully in a moment, it would appear that the chief principle of the surgical treatment of actinomycosis is to remove as much of the disease as possible, and to *establish a free channel from the seat of the disease through the skin so as to make free external drainage.* This, besides *aiding the action of the iodide of potassium,* diminishes the danger of swallowing and inspiring the actinomyces, and prevents the organism finding its way into other portions of the body. This principle of the surgical treatment applies wherever the disease is located.

The death rate of reported cases of actinomycosis in the United States is very high—47 per cent.; and the use of iodide of potassium in these cases is very low—only about 10 per cent. This fact is the main reason that induced me to make this contribution, for it appears that we have not yet, in this country at least, learned the value of iodide of potassium in this disease.

It is very probable that human actinomycosis has a strong natural tendency to get well of itself, especially when situated in favorable locations and when simple sinuses have been naturally opened, or have been opened with the knife, establishing drainage. But, as the cases in the United States clearly show, a large percentage will die when no other than surgical aid is given.

Dr. Victor Leiblein reports from Prof. Wölfler's clinic, Prag, in "Beiträge zur Klinischen Chirurgie, 28th vol.,—first part, 1900,"—a careful review of the iodide of potassium treatment of Human Actinomycosis. He gives the following table: Head and throat, 49 cases, 36 recovered; 6 improved; 4, treatment not finished, and 3 only died. Lungs, 5 cases treated, 2 recovered; 1, not improved; 2 died. Intestine, 6 treated, 4 recovered, 1 improved, 1 died. Region not mentioned, 2 treated, both improved. This in very striking contrast to the cases reported from the United States.

In one of the cases of actinomycosis of the lung reported by Leiblein, my own, No. 1, as reported here, I know the iodide of potassium was not thoroughly used. Certainly not as I would use it to-day; and many of his other cases, also, had the iodide of potassium used in an imperfect manner.

All of which would clearly indicate that a larger percentage of cures could have been obtained had the best method of using iodide of potassium been employed.

To emphasize the importance of surgical means, it is worth while to note that the iodide of potassium *does not destroy the parasite,* although the iodine compounds have been found in the pus from the actinomycotic lesions.

It will be seen from Leiblein's table that the cases involving the head and the throat are very much more amenable to treatment than are the cases involving the lungs. The intestines furnish a suitable location for the treatment by the iodide of potassium.

It is very important to give Leiblein's opinion as to the action of the iodide of potassium. He thinks its favorable action is due to the disintegration of the cellular infiltration, and its discharge through the fistulos tracts, thus facilitating the discharge of the parasites.

This furnishes an explanation why throat and intestine cases do better than lung cases. In the first two, drainage is more easily established. In the last, it is more difficult, or impossible for drainage to be effectual.

The advent of mixed infection retards, and even wholly prevents the favorable action of iodide of potassium; and, in acute, phlegmonous cases the iodide of potassium acts too slowly to be of very much benefit. Also, in cases very much reduced in strength, the iodide of potassium may act too slowly to prevent a fatal issue.

Of great interest are Leiblein's observations on the use of iodide of potassium, after unsuccessful operative interference. Once death supervened, once marked improvement, and three times cured. One of the last group was twice ineffectually operated upon. The iodide was used three times, and yet operations were necessary; but in these cases the iodide of potassium was not used sufficiently long.

*Method of Giving Iodide of Potassium.*—Prof. Cary, of Auburn, Ala., who has had a good deal of experience in treating actinomycotic cattle, informs me that the beneficial result of the iodide of potassium is not obtained until iodism is produced.

With this in view, it was our purpose to produce some iodism in case No. 2, reported here, which has recovered. But, beyond the merest signs, no iodism resulted, although the patient took 600 grains of iodide of potassium per day for weeks at a time.

In the open sinuses, in this case, I could not find the parasite, but one of the last manifestations of the actinomycotic process in this patient's neck was a small abscess, which I opened, and which discharged about one-half dram of characteristic pus, containing numerous large, healthy-looking actinomycotic parasites. It is very interesting that in this case there was no marked improvement under the iodide of potash until external drainage was established.

This adds confirmation to the statement that the iodide of potassium does not kill the parasite, but acts by facilitating its discharge.

The iodide of potassium, to be serviceable, must be given for several months, or longer. From a study of the cases cured by the iodide of potassium, it appears that 60 or 100 grains a day is sufficient.

In my opinion, if the case does not rapidly improve, an effort should be made to get the physiological action of the drug.

A ten per cent. solution of iodide of potassium gauze should be used in packing the cavities and sinuses, and in dressing the wounds.

The treatment of human actinomycosis is surgical—the removal of as much of the disease as possible, *and the establishment of free external drainage;* and medical, the use of iodide of potassium *to bring about the solution of the cellular infiltration and the discharge of the parasite.*

## Figures and Tables

**Plate No. 1. f1:**
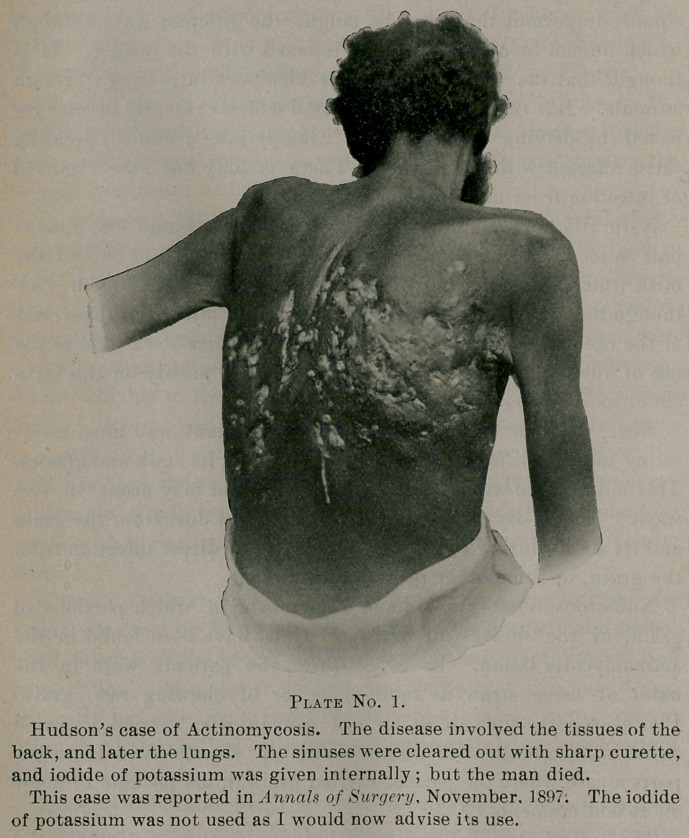


**Plate No. 2. f2:**